# A Novel Candidate Gene Associated With Body Weight in the Pacific White Shrimp *Litopenaeus vannamei*

**DOI:** 10.3389/fgene.2019.00520

**Published:** 2019-05-31

**Authors:** Quanchao Wang, Yang Yu, Qian Zhang, Xiaojun Zhang, Jianbo Yuan, Hao Huang, Jianhai Xiang, Fuhua Li

**Affiliations:** ^1^Key Laboratory of Experimental Marine Biology, Institute of Oceanology, Chinese Academy of Sciences, Qingdao, China; ^2^Laboratory for Marine Biology and Biotechnology, Qingdao National Laboratory for Marine Science and Technology, Qingdao, China; ^3^University of Chinese Academy of Sciences, Beijing, China; ^4^Hainan Grand Suntop Ocean Breeding Co., Ltd., Wenchang, China; ^5^Center for Ocean Mega-Science, Chinese Academy of Sciences, Qingdao, China

**Keywords:** penaeid shrimp, growth traits, GWAS, candidate gene, class C scavenger receptor

## Abstract

Improvements of growth traits are always the focus in selective breeding programs for the Pacific white shrimp *Litopenaeus vannamei* (*L. vannamei*). Identification of growth-related genes or markers can contribute to the application of modern breeding technologies, and thus accelerate the genetic improvement of growth traits. The aim of this study was to identify the genes and molecular markers associated with the growth traits of *L. vannamei*. A population of 200 individuals was genotyped using 2b-RAD techniques for genome-wide linkage disequilibrium (LD) analysis and genome-wide association study (GWAS). The results showed that the LD decayed fast in the studied population, which suggest that it is feasible to fine map the growth-related genes with GWAS in *L. vannamei*. One gene designated as *LvSRC*, encoding the class C scavenger receptor (SRC), was identified as a growth-related candidate gene by GWAS. Further targeted sequencing of the candidate gene in another population of 322 shrimps revealed that several non-synonymous mutations within *LvSRC* were significantly associated with the body weight (*P* < 0.01), and the most significant marker (SRC_24) located in the candidate gene could explain 13% of phenotypic variance. The current results provide not only molecular markers for genetic improvement in *L. vannamei*, but also new insights for understanding the growth regulation mechanism in penaeid shrimp.

## Introduction

*Litopenaeus vannamei* (*L. vannamei*), as one of the most economically important marine aquaculture species, is playing an important role in fulfilling the increased requirement for high quality animal proteins consumption. It is estimated that *L. vannamei* provided approximately 70% of the total shrimp production in the world (Li et al., 2018). The continuous development of shrimp industry drives the genetic improvement of important economic traits. During the past decade, large efforts have been put to improve the key economic traits, including growth traits and disease resistance (Argue et al., 2002; Huang et al., 2011; Andriantahina et al., 2013). Among these traits, growth is always the focus of breeders because it directly contributes to the shrimp production. In general, the growth trait, such as body weight, presents a moderate to high heritability (Sui et al., 2016; Nolasco-Alzaga et al., 2017), and the genetic gain per generation reached 10.7% (Andriantahina et al., 2012), which is higher than that of farmed terrestrial species. At present, broodstocks with high and stable growth traits are urgently needed to meet the requirement of shrimp culture industry. Modern molecular breeding technologies, including marker assisted selection (MAS), gene assisted selection (GAS), and gene editing technology, etc., are promising methods for accelerating the genetic improvement of growth traits (Gjedrem and Baranski, 2010). Till present, several growth-related genes involved in molting and muscle development such as molt-inhibiting hormone (MIH), crustacean hyperglycaemic hormone (CHH), ecdysteroid receptor (EcR), actin and myostatin differential factor 11 (MSTN), etc., were identified (Li et al., 2011; Jung et al., 2013). However, the growth traits are likely to be highly polygenic, and the underlying physiological bases may involve complex regulatory networks of many interacting genes with different effects. Although QTL mapping analysis of growth traits has been conducted in *L. vannamei* (Yu et al., 2015), limited number of markers makes the fine mapping of QTLs difficult. Hence, new methods are urgently required to localized the major genes or markers related to growth traits in shrimp.

Genome-wide association study (GWAS) have been successfully performed to identify genes participating in the regulation of complex traits in human (Mccarthy et al., 2008), livestock (Zhang et al., 2013), and crop (Huang and Han, 2014). Recently, with the development of high-throughput sequencing technologies and the successive decoding of aquatic animal genomes, GWAS is becoming a powerful tool to analyze the genetic basis of complex traits, and some candidate genes associated with growth traits or disease resistance were reported in a number of aquatic animals, including Atlantic salmon (Sodeland et al., 2013; Gutierrez et al., 2015; Correa et al., 2017), rainbow trout (Vallejo et al., 2014; Gonzalez-Pena et al., 2016), and catfish (Geng et al., 2015; Jin et al., 2016). However, there is no relevant study in *L. vannamei*. In the present study, we aimed to identify growth-related loci or genes in diverse population by using GWAS integrated with candidate gene association study, and provide a convinced result for revealing the molecular mechanism of growth traits in *L. vannamei*.

## Materials and Methods

### Animals and Genotyping

Two populations, designated as A16 and B2016_13, had been used in this study. These two populations were created and cultured at Guangtai Marine Breeding Company in Hainan province, China. The population A16 was established in 2015 as previously described (Wang et al., 2017). Briefly, it was composed of 200 individuals from 13 full-sib families (offsprings of 13 dams and 13 sires). Each full-sib family was cultured separately in the 5 m^2^ tank before their body length reached 3 cm, and then 50 individuals from each family were transferred to a 10 m^2^ pond for culture. At the harvest, two hundred individuals were randomly collected for the phenotyping and genotyping. For population B2016_13, it was constructed in 2016 and the individuals from multiple full-sib families were mixed after spawn, a total of 322 individuals were collected and phenotyped. The sex of all individuals from these two populations was determined by sex-associated marker (Yu et al., 2017). The average body weight for A16 population was 5.56 ± 2.16 g and that for B2016_13 population was 9.51 ± 3.30 g.

Total DNA of each sample was extracted from the muscle of shrimp using Plant Genomic DNA Kit (TIANGEN, Beijing, China) according to the manual instruction. The purity and integrity of the extracted DNA was determined by using a NanoDrop 1000 Spectrophotometer (NanoDrop, Wilmington, DE, United States) and electrophoresis on 1% agarose gel. Qualified genomic DNA was stored at -20°C.

All individuals from A16 population were genome-widely genotyped using 2b-RAD method (Wang et al., 2012), which was carried out by OE Biotech Company (OE Biotech, Shanghai, China). The reference genome were *de novo* assembled using the reads from the 10 individuals with high sequencing depth, and the genotyping of each individuals were conducted using RADtyping program (Fu et al., 2013). The shrimp from B2016_13 population were genotyped for the targeted locus of the candidate genes by using PCR-based sequencing.

### Genome-Wide Linkage Disequilibrium Analysis

The physical position of SNPs was identified by blasting the 2b-RAD marker to the assembled reference genome of *L. vannamei* (Zhang et al., 2019). LD was estimated by using SNPs genotyping and physical position information. The squared correlation of allele frequencies (r^2^) was used as a measure of LD (Hill, 1974). The r^2^ between each pair of SNPs on the same chromosome was calculated using “genetics” package in R (R Core Team, 2018). The decay of the r^2^ with distance was fitted using the expected value of r^2^ under drift-recombination equilibrium that had previously been implemented (Remington et al., 2001; Marroni et al., 2011).

### Genome-Wide Association Study

Genome-wide association study for body weight were performed using the *egscore* function in the R package GenABEL (Aulchenko et al., 2007). The potential bias in association caused by hidden population stratification was corrected by principal components (PCs) of genomic kinship matrix (Price et al., 2006). Via inspecting the eigenvalues of the kinship matrix, the first four PCs were selected to adjusting the genotypes and phenotypes. Sex was selected as fixed factor. Besides, adjusting with PCs did not remove all population stratification, hence a further genomic control correction of the obtained *P* values was performed using the inflation factor. Considering the small sample population size and the sparse marker density, the significance level for genome-wide significance was set as *P* = 0.01 ($-\log_{10}^{p}\;$ = 2).

### Candidate Genes Study

**FIGURE 1:**
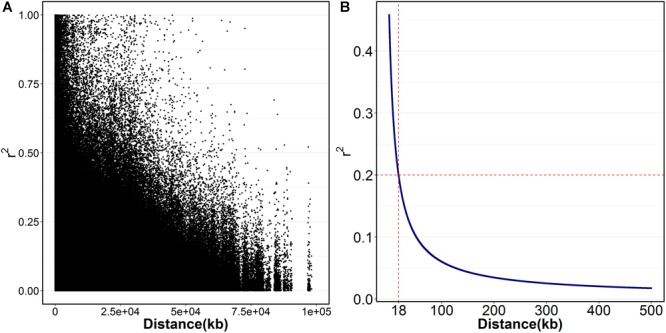
The extent of LD (r^2^) in *Litopenaeus vannamei*. **(A)** The scatter plot of r^2^ against the physical distance; **(B)** the fitted decay curve of r^2^ within 500 kb.

The sequences of SNPs associated with body weight were compared by BLAST against the genome sequence of the *L. vannamei* (Zhang et al., 2019). Given the rapid LD decay rate (Figure 1), the genes within the 18 kb upstream and downstream of the significant SNPs were considered as candidate genes. Then, the SNPs in the coding region of candidate genes were detected by PCR-based sequencing. The non-synonymous SNPs were genotyped in all the individuals from B2016_13 population and tested for association with body weight. The association test of candidate genes was performed by using linear model in R software, and sex was selected as covariate. According to the principle of variance decomposition in linear model (Ho and Lin, 2003), the ratio of phenotypic variance (Var) explained by the SNP, significantly associated with the body weight of *L. vannamei*, was calculated as following:

$$\begin{array}{c} Var=\frac{SS_{R}}{SS_{R}+SS_{S}+SS_{E}}\times 100\% \end{array}$$

where SS_R_ is the sum of squares produced by the SNP; SS_S_ is the sum of squares produced by the sex; SS_E_ is the residual sum of squares.

## Results

### Genome-Wide Linkage Disequilibrium

A total of 23,049 single nucleotide polymorphism (SNP) markers were obtained after quality control that SNPs with missing rate at more than 5% across samples and minor allele frequency less than 0.05 were removed. By blasting these markers to the assembled reference genome of *L. vannamei*, 13,814 SNPs were successfully mapped onto chromosomes. These SNPs were located on 44 chromosomes (Chrs) with a median distance between adjacent markers of 226.12 kb and an average of 314 SNP markers per chromosome (Table 1). The number of SNPs varied among Chrs, from 43 on chr14 to 761 on chr1. The average distance between the adjacent SNPs pairs within Chr was also different, ranging from 91.45 kb on chr15 to 1431.73 kb on chr39. A total of 2,579,595 paired SNPs had been used to calculate the r^2^. The r^2^ with distance was plotted in Figure 1A. The overall LD across the genome between all paired SNPs was 0.06 and only few values (0.4%) of *r*^2^ > 0.6 were found. A rapid decay of LD was presented in Figure 1B, where r^2^ decreased to 0.2 at SNP marker interval of 18 kb.

**Table 1:** Numbers of SNPs and the average distances between adjacent SNP pairs for each chromosome.

Chr	No. of SNPs	Average distance (kb)	Chr	No. of SNPs	Average distance (kb)
chr1	761	129.27	chr23	121	392.39
chr2	234	181.74	chr24	213	257.15
chr3	488	109.82	chr25	378	151.94
chr4	284	237.89	chr26	320	199.39
chr5	259	161.81	chr27	363	160.89
chr6	304	225.06	chr28	373	174.65
chr7	245	202.13	chr29	388	192.25
chr8	155	474.03	chr30	183	339.49
chr9	531	102.06	chr31	370	182.45
chr10	381	138.56	chr32	303	200.49
chr11	346	112.84	chr33	191	131.57
chr12	200	249.82	chr34	437	160.01
chr13	492	110.15	chr35	159	411.33
chr14	43	223.06	chr36	243	162.66
chr15	517	91.45	chr37	263	228.44
chr16	448	131.59	chr38	195	370.07
chr17	503	117.04	chr39	56	1431.73
chr18	283	186.67	chr40	283	204.97
chr19	482	114.79	chr41	201	247.52
chr20	550	127.01	chr42	350	185.81
chr21	96	113.30	chr43	269	226.41
chr22	250	199.09	chr44	303	198.62

*Chr, chromosome; No, number.*

### Genome-Wide Association Study

**FIGURE 2:**
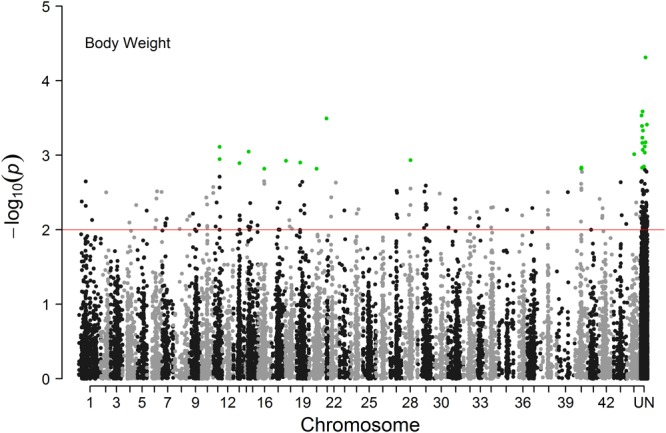
Manhattan plot of genome-wide association analysis for body weight of *Litopenaeus vannamei*.

The Manhattan plot of all SNPs is shown in Figure 2. A total of 226 SNPs significantly associated with body weight were identified at a threshold of *P* < 0.01 ($-\log_{10}^{p}\;$ > 2). Among the 226 significant SNPs, 84 SNPs are currently unassigned to chromosomes, and the remaining 142 SNPs were successfully mapped to 39 chromosomes. Given the large number of significant markers, the first twenty significant markers were used for subsequent analysis. Of these, 12 SNPs are currently unassigned to chromosomes, and the remaining 8 SNPs were successfully mapped to 6 chromosomes (Table 2). Gene annotation showed that only the marker ref-613798-25 was located in the coding region of one gene which can encode the class C scavenger receptor (SRC). Therefore, the gene, referred to hereafter as *LvSRC*, was considered as the most likely candidate gene for body weight in *L. vannamei*.

**Table 2:** Summary of the first twenty significant SNPs associated with body weight by GWAS.

Marker ID	Chr	Alleles	MAF	*P* value
ref-466132-2	UN	C/G	0.30	4.85E-05
ref-123254-9	UN	T/A	0.10	2.59E-04
ref-231-26	UN	T/C	0.09	2.91E-04
ref-331386-1	21	G/C	0.14	3.20E-04
ref-331386-3	21	C/G	0.14	3.20E-04
ref-628970-14	UN	C/T	0.31	3.88E-04
ref-53799-25	UN	G/A	0.15	4.05E-04
ref-165277-22	UN	T/C	0.35	4.67E-04
ref-97619-15	UN	A/G	0.06	5.80E-04
ref-456266-7	UN	A/G	0.09	6.69E-04
ref-85081-3	UN	C/G	0.09	6.83E-04
ref-372581-7	UN	T/A	0.31	7.61E-04
ref-382704-3	11	C/A	0.08	7.71E-04
ref-150968-7	UN	T/C	0.16	8.43E-04
ref-249669-5	15	T/C	0.17	8.91E-04
ref-377367-16	UN	G/A	0.14	9.21E-04
ref-613742-15	44	C/G	0.49	9.67E-04
ref-595037-3	11	G/T	0.09	1.13E-03
ref-58593-5	28	A/C	0.08	1.17E-03
ref-613798-25	18	A/G	0.19	1.18E-03

*Chr, chromosome; MAF, minor allele frequency; and UN, no chromosome was assigned for the marker.*

### Candidate Gene Association Study

**FIGURE 3:**
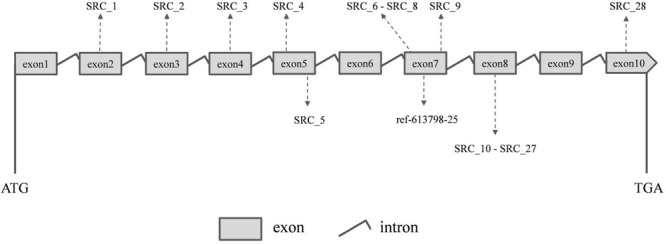
The positions of identified SNPs in the *LvSRC* gene.

Ten PCR primers (Table 3) were developed from the targeted genome sequences of *LvSRC* and then used to amplify for a specific locus. A total of 29 SNPs (including ref-613798-25) were identified in the coding region of *LvSRC* (Figure 3). Among these, 20 SNPs are synonymous mutation (Supplementary Table S1), and 9 SNPs are non-synonymous mutations (Table 4). All these non-synonymous mutations were examined for association with body weight in B2016_13 population. The statistical results showed that 7 SNPs presented significant association (*P* < 0.01) with body weight, and the SNP (SRC_24) contributed most significantly to the trait and it could explain 13% of phenotypic variance, followed by SRC_13 (6%), SRC_15 (6%), SRC_7 (6%), SRC_14 (6%), ref-613798-25 (4%), and SRC_27 (4%).

**Table 3:** The primers designed for the identification of SNP in the coding region of *LvSRC*.

Primer ID	Primer sequence (5′–3′)		Fragment length (bp)	Ta (°C)
Primer0	Forward	GTCTGTTGGAGTGGTAGCGTTTT	281	58
	Reverse	CCAGCACATCTACGCCTTCC		
Primer1	Forward	TCTTCCACATCCACCAACGC	210	58
	Reverse	TCCCTCCCCAGAAAAGAAGC		
Primer2	Forward	CATTCCAGTTGATGCCGTCG	177	58
	Reverse	CATTCTCACCCTCCCGTTCC		
Primer3	Forward	GCCCATCTCAACTCACCTTCG	322	58
	Reverse	GGCAACCTTTTCACCTCCCTA		
Primer4	Forward	CCAAGCCTCGTGAACCGTAG	294	58
	Reverse	CGCTGACCCTGACATAGTGC		
Primer5	Forward	TGGTCGTAGTGGTCTCAGTATCG	168	58
	Reverse	CTGTGAGCGTGTTCCGTCTC		
Primer6	Forward	GTCGTAACAGGTGACCCATCG	142	58
	Reverse	CCAGCACATCTACGCCTTCC		
Primer7	Forward	TCTTGTTTTCCGTTCCCTCG	446	58
	Reverse	CAGCACATCTACGCCTTCCAC		
Primer8	Forward	CCGTATTTGTTGAATGAGTGGG	339	58
	Reverse	CTACCACTCCAACAGACGAAGG		
Primer9	Forward	GTGCTCCCCAGGCTGAAGAT	174	58
	Reverse	CATCTCCCTGGTCGTCTTCG		

*Ta, the optimal annealing temperature.*

**Table 4:** Summary of the non-synonymous SNPs in the coding region of *LvSRC* gene.

Marker ID	Primer ID	Site	Alleles	*P* value	Var (%)
ref-613798-25	Primer0	exon7	A/G	1.27E-03	4
SRC_7	Primer6	exon7	A/G	1.61E-04	6
SRC_8	Primer6	exon7	T/C	9.10E-02	NA
SRC_11	Primer7	exon8	A/C	1.10E-01	NA
SRC_13	Primer7	exon8	T/C	1.01E-04	6
SRC_14	Primer7	exon8	A/C	1.89E-04	6
SRC_15	Primer7	exon8	A/G	1.03E-04	6
SRC_24	Primer8	exon8	A/C	1.31E-10	13
SRC_27	Primer8	exon8	T/A	6.45E-03	4

*Var, the ratio of phenotypic variance explained by the SNP; NA, null value.*

## Discussion

To our knowledge, this is the first report about LD pattern and GWAS in *L. vannamei.* Overall, the decay of LD in this population is rapid, which suggested it is feasible to perform the fine mapping of growth-related genes with GWAS. However, it is worth noting that high-density markers will be required to increase the power of GWAS. An average r^2^ greater than 0.2 has been proposed to be the desirable requirement for GWAS in previous studies (Meuwissen et al., 2001; Mckay et al., 2007). Considering a genome length of 2.64 Gb in *L. vannamei*(Yu et al., 2015), ∼150 K fully informative markers would be needed to saturate the requirement of GWAS at an average resolution of 18 kb.

Although only ∼23 K markers were used for GWAS in this study, a large number of markers significantly associated with body weight were identified (*P* < 0.01). This result may confirm the previous speculation that the shrimp growth is highly polygenic, and regulated by complex regulatory networks of many interacting genes (Moss and Moss, 2009). The current identified *LvSRC* gene may be one of those interacting genes and play an important role in the regulation of shrimp growth.

Scavenger receptors (SRs) comprise a large family of structurally diverse transmembrane cell surface glycoproteins and nine heterogeneous subclasses (A-I) were classified in accordance with their multidomain structures (Canton et al., 2013). As one member of SRs, SRC has only been identified in a few invertebrates, including *Drosophila melanogaster, Aedes aegypti*, and *Marsupenaeus japonicus*. Especially, previous studies only reveal the function of SRC in immunological process (Rämet et al., 2001; Yang et al., 2016, 2017), and whether SRC participates in growth regulation remains largely unknown. Indeed, although SRs family encompasses a wide range of molecules with little structural homology (Canton et al., 2013), almost all of them have been characterized in vertebrates by the common feature to bind modified low density lipoproteins (LDLs), such as oxidized LDL (OxLDL) and acetylated LDL (AcLDL). Therefore, SRs can play a central role in lipid metabolism. The similar function of SRs was also revealed in invertebrates. For example, in *Macrobrachium nipponense*, the expression of gene encoding the class B SR can be regulated by dietary lipid sources including soybean and linseed oils (Ding et al., 2016). Therefore, it’s interesting to note that SRC may be related to the body weight of shrimp by participating in lipid metabolism.

The significant SNPs in the coding region of *LvSRC*, especially the marker SRC_24, could be promising candidates for marker assisted breeding of growth traits in *L. vannamei*. Nevertheless, it is still uncertain that which mutations within the *LvSRC* gene are the causative loci associated with growth of shrimp. Therefore, gene editing technology will be a powerful tool to determine the causative locus in the future. Besides, it is important to note that the phenotypic variation of complex traits can be affected by the mutations in the non-coding region of genes, including untranslated region (Si et al., 2016) or promoter region (Wang et al., 2016). Therefore, it should be further investigated that whether the causative loci located in the non-coding region of *LvSRC*.

In addition, it is important to note that a number of significant markers from GWAS failed to be annotated. There may be two reasons for this result. Firstly, parts of the reference genome was not fully assembled which result in the difficulty of gene annotation. Secondly, the region of candidate genes was determined based on the average LD decay rate in this study; however, the LD decay of different genomic regions might be quite different (Lu et al., 2012; Kawakami et al., 2017). Therefore, in the future, more growth-related genes would be revealed with the increase of genome information and the detail survey of LD decay of different genome regions.

## Conclusion

In this study, the LD decay of the studied population is rapid with an average *r*^2^ (0.2) values at 18 kb, which suggested that it is feasible to fine map the growth-related genes by using this population. By using GWAS integrated with candidate gene association study, the *LvSRC* was proved to be associated with growth traits. This result not only provides molecular markers that may contribute to accelerate the genetic improvement for penaeid shrimp, but also provides new insights to help understand regulatory mechanism of shrimp growth. Further studies are needed to fine mapping the causative mutation in the *LvSRC* and investigate its regulatory mechanism on shrimp growth.

## Author Contributions

QW and YY conducted the experiment and data processing. JX and FL conceived and supervised the project. QZ, XZ, and JY contributed to prepare the genomic DNA for SNP genotyping. HH prepared and cultured the experimental animals. QW, YY, and FL wrote the manuscript. All authors have read and approved the manuscript.

## Conflict of Interest Statement

HH was employed by company Hainan Grand Suntop Ocean Breeding Co., Ltd. The remaining authors declare that the research was conducted in the absence of any commercial or financial relationships that could be construed as a potential conflict of interest.
