# Supplementary material for: A Novel Candidate Gene Associated With Body Weight in the Pacific White Shrimp Litopenaeus vannamei
Source: Front Genet. 2019 May 31;10:520. doi: 10.3389/fgene.2019.00520 (PMC6555256; doi:10.3389/fgene.2019.00520)
Supplement: Supplementary file 1 [file Table_1.docx]

Supplemental table S1 | Summary of the synonymous SNPs in the coding region of *LvSRC* gene

| Marker ID | Primer ID | Site | Alleles |
| --- | --- | --- | --- |
| SRC_1 | Primer1 | exon2 | T/C |
| SRC_2 | Primer2 | exon3 | G/C |
| SRC_3 | Primer3 | exon4 | G/C |
| SRC_4 | Primer4 | exon5 | G/C |
| SRC_5 | Primer4 | exon5 | C/T |
| SRC_6 | Primer5 | exon7 | T/C |
| SRC_9 | Primer6 | exon7 | T/A |
| SRC_10 | Primer7 | exon8 | G/C |
| SRC_12 | Primer7 | exon8 | A/C |
| SRC_16 | Primer7 | exon8 | C/T |
| SRC_17 | Primer7 | exon8 | A/C |
| SRC_18 | Primer7 | exon8 | G/A |
| SRC_19 | Primer7 | exon8 | C/T |
| SRC_20 | Primer7 | exon8 | C/T |
| SRC_21 | Primer7 | exon8 | C/G |
| SRC_22 | Primer7 | exon8 | T/G |
| SRC_23 | Primer7 | exon8 | G/A |
| SRC_25 | Primer8 | exon8 | T/C |
| SRC_26 | Primer8 | exon8 | G/C |
| SRC_28 | Primer9 | exon10 | T/C |
